# *Vibrio vulnificus* RtxA1 cytotoxin targets filamin A to regulate PAK1- and MAPK-dependent cytoskeleton reorganization and cell death

**DOI:** 10.1080/22221751.2019.1632153

**Published:** 2019-06-25

**Authors:** Rui Hong Guo, Young Jun Im, Soo Im Shin, Kwangjoon Jeong, Joon Haeng Rhee, Young Ran Kim

**Affiliations:** a College of Pharmacy and Research Institute of Drug Development, Chonnam National University, Gwangju, Republic of Korea; b Clinical Vaccine R&D Center and Department of Microbiology, Chonnam National University Medical School, Hwasun, Republic of Korea; c Department of Bioengineering and Biotechnology, College of Engineering, Chonnam National University, Gwangju, Republic of Korea

**Keywords:** RtxA1 toxin, filamin, pak1, JNK, p38, *V. vulnificus*, host–parasite interaction

## Abstract

Cytoskeletal rearrangement and acute cytotoxicity occur in *Vibrio vulnificus-*infected host cells. RtxA1 toxin, a multifunctional autoprocessing repeats-in-toxin (MARTX), is essential for the pathogenesis of *V. vulnificus* and the programmed necrotic cell death. In this study, HeLa cells expressing RtxA1 amino acids 1491–1971 fused to GFP were observed to be rounded. Through yeast two-hybrid screening and subsequent immunoprecipitation validation assays, we confirmed the specific binding of a RtxA1_1491–1971_ fragment with host-cell filamin A, an actin cross-linking scaffold protein. Downregulation of filamin A expression decreased the cytotoxicity of RtxA1 toward host cells. Furthermore, the phosphorylation of JNK and p38 MAPKs was induced by the RtxA1-filamin A interaction during the toxin-mediated cell death. However, the phosphorylation of these MAPKs was not observed during the RtxA1 intoxication of filamin A-deficient M2 cells. In addition, the depletion of pak1, which appeared to be activated by the RtxA1-filamin A interaction, inhibited RtxA1-induced phosphorylation of JNK and p38, and the cells treated with a pak1 inhibitor exhibited decreased RtxA1-mediated cytoskeletal rearrangement and cytotoxicity. Thus, the binding of filamin A by the RtxA1_1491–1971_ domain appears to be a requisite to pak1-mediated MAPK activation, which contributes to the cytoskeletal reorganization and host cell death.

## Introduction

*Vibrio vulnificus* is an opportunistic human pathogen that causes fatal septicemia and necrotic wound infections, which results in deaths within a few days [1]. RtxA1 toxin is a multifunctional autoprocessing repeats-in-toxin (MARTX) that plays an essential role in the pathogenesis of *V. vulnificus* and is involved in the programmed necrotic death of host cells [2–5]. RtxA1 is responsible for cytoskeletal rearrangement, contact cytotoxicity, hemolysis, tissue invasion, and lethality in mice [3,6,7] and has numerous functional regions. Conserved N- and C-terminal regions of the *V. vulnificus* MARTX toxin form pores in eukaryotic cell membranes and are essential for the delivery of effector domains from bacteria to the host cell cytosol, as well as for promoting cell lysis [8,9]. The central effector domain region of RtxA1 causes biphasic epithelial barrier disruption and systemic spread from the intestine, while the cysteine protease domain (CPD) is essential for toxin autoprocessing [10,11]. Previous studies have reported that the actin cross-linking domain (ACD) of the *Vibrio cholerae* MARTX toxin is responsible for the rapid cell rounding observed to occur in response to this protein through catalyzing the formation of an intermolecular iso-peptide bond located in the hydrophobic and the DNaseI-binding loops of actin [12]. Furthermore, ACD-induced actin oligomers have been shown to disrupt the action of the major actin assembly proteins, formins, which control actin polymerization [13]. Although *V. vulnificus* RtxA1 is highly homologous to the *V. cholerae* MARTX toxin and causes actin aggregation [7], the biotype 1 MARTX of the *V. vulnificus* CMCP6 and MO6-24/O strains lacks the ACD [5,9], suggesting that other actin-regulatory proteins may be involved in the cytoskeletal rearrangements caused by RtxA1 from the *V. vulnificus* biotype 1 MO6-24/O strain. Potential candidates are the Rho guanosine triphosphatase (GTPase) inactivation domain (RID) or the Ras/Rap1-specific endopeptidase RRSP (formerly DUF5), both of which have been shown to induce cell rounding through ectopic expression studies. However, the *V. vulnificus* biotype 1 MO6-24/O strain does not have an RRSP domain [14–16]. A recent report showed that a conserved effector domain of the *V. vulnificus* MARTX toxin, RID, could mediate the lysine N^ϵ^-fatty acyltransferase activity toward Rho GTPases and promote cell rounding by disrupting the host actin cytoskeleton [17]. In addition, other domains of unknown function may contribute to modulate the cytoskeleton. Still much is remained obscure how RTX toxins induce cytoskeletal rearrangements by interacting with host factors. Previously, we reported that prohibitin is a host partner of *V. vulnificus* RtxA1 [6]. In this study, a fragment of the conserved N-terminal domain of RtxA1 toxin (corresponding to RtxA1 amino acids 1491–1971 of *V. vulnificus* 29307), named RtxA1_1491–1971_, was investigated. Interestingly, RtxA1_1491–1971_ is approximately 25% identical with ezrin, radixin, moesin (ERM) family proteins that function as linkers between the plasma membrane and actin cytoskeleton [18]. ERM family proteins have also been reported to be involved in virus-induced cytoskeleton rearrangement of host cells [19,20]. We observed that HeLa cells expressing RtxA1_1491–1971_ fused to GFP became rounded. We hypothesized that this region may play a role in the cytoskeletal rearrangement caused by RtxA1. In this study, we performed a yeast two-hybrid screening assay to identify host factors that specifically interact with RtxA1_1491–1971_, resulting in the putative identification of filamin A, an actin cross-linking scaffold protein acting as a host partner. We show that *V. vulnificus* RtxA1_1491–1971_ specifically interacts with filamin A, contributing to cytoskeletal rearrangement and acute necrotic cell death.

## Materials and methods

### Cell cultures and reagents

The clinical isolate *V. vulnificus* MO6-24/O wild-type (wt), the *rtxA1* mutant CMM744 (*rtxA1^−^*), and the complement strain of *rtxA1^−^* CMM745 were used in this study [6]. Bacteria were inoculated in 0.9% NaCl heart infusion (HI) broth (BD, MD, USA) and grown at 37°C shaking at 200 rpm. To prepare a log-phase culture of *V. vulnificus*, bacteria cultured overnight were diluted 200-fold in fresh HI broth and cultured for another 4 h.

HeLa cells (Korea Cell Line Bank, Seoul, Korea) were cultured in Dulbecco’s modified Eagle’s medium (DMEM; Welgene, Korea) supplemented with 10% heat-inactivated fetal bovine serum (FBS; Thermofisher Scientific, MA, USA) at 37°C in an incubator with 5% CO_2_. M2 cells (a human melanoma cell line lacking filamin) and A7 cells (an M2 subline, which were stably transfected with a full-length filamin cDNA) (ATCC, VA, USA) were maintained in DMEM containing 8% newborn calf serum and 2% FBS. A7 cells were cultured in the presence of 500 µg/mL G418 (Thermofisher Scientific, MA, USA) and 10 mM HEPES (Sigma chemical, MO, USA) to maintain filamin expression.

Pharmacological antagonists, such as SP600125 (10 µM, a JNK antagonist), SB203580 (10 µM, a p38 MAPK inhibitor), IPA-3 (10 µM, a pak1 inhibitor), and PD98059 (10 µM, an ERK inhibitor) were purchased from Selleckchem (Houston, TX, USA).

### Anti-RtxA1_1491–1971_ antibody production

A DNA fragment encoding the RtxA1_1491–1971_ domain was PCR amplified and cloned into the expression vector pGEX-4T1 (Amersham Pharmacia Biotech Inc., Piscataway, NJ). The primers used in this study are listed in Table 1. The glutathione *S*-transferase (GST) fusion protein was purified by affinity chromatography following the manufacturer’s recommendations (Amersham Pharmacia Biotech Inc.). A polyclonal antibody against the GST-RtxA1_1491–1971_ fusion protein was raised in New Zealand white rabbits using a previously described method [6], and the antibody was thoroughly absorbed using *rtxA1* mutant bacterial lysates and HeLa lysates, as described previously [21].

**Table 1.** Primers used in PCR analysis.

	Primers	Sequences
**pGEX-4T1::*rtxA1*_1491–1971_**	pGEX-4T1::*rtxA1*_1491–1971_-F	5′-CGG GAT CCT ATG GCG TGA ACG GCG AAG-3′
	pGEX-4T1::*rtxA1*_1491–1971_-R	5′-CGG GAT CCA GCA GCC ACA AGC GAT TC-3′
**pEGFP::*rtxA1*_1491–1971_**	pEGFP::*rtxA1*_1491–1971_-F	5′-GG ATC CTC TAT GGC GTG AAC GGC GAA-3′
	pEGFP::*rtxA1*_1491–1971_-R	5′-CGA GCA GCC ACA AGC GAT TC-3′

### GFP-RtxA1_1491–1971_ fusion protein expression in HeLa cells

A DNA fragment encoding RtxA1_1491–1971_ was PCR amplified with native Pfu DNA polymerase (Takara, Tokyo, Japan) and cloned into the expression vector pEGFP-C1 (Clontech, Palo Alto, CA, USA). The primers used are listed in Table 1. The resulting construct (pEGFP::*rtxA1*_1491–1971_) was transfected into HeLa cells overnight using Lipofectamine 3000 (Thermofisher Scientific, MA, USA).

### Immunostaining

HeLa cells seeded into 8-well chamber plates (Nalge Nunc International, New York, USA) were infected with *V. vulnificus* strains at an MOI of 100, after which cells were fixed in 3.7% formaldehyde (Thermofisher Scientific, MA, USA) for 10 min, permeabilized with 0.1% Triton X-100 (Sigma-Aldrich, MO, USA), and incubated in a blocking solution for 30 min. Cells were then incubated for 1 h with anti-RtxA1_1491–1971_ rabbit polyclonal antibody and anti-filamin A mouse monoclonal antibody. Subsequently, cells were labelled with FITC-conjugated anti-rabbit (Sigma, MO, USA) and Texas Red-conjugated anti-mouse secondary antibodies (Molecular Probes) for 1 h, and then were mounted with an anti-fade reagent with DAPI (Thermofisher Scientific, MA, USA). Confocal images were acquired using a laser scanning confocal microscope (Leica Microsystems TCS NT, Leica, Germany) at the Korea Basic Science Institute (KBSI, Gwangju, Korea).

HeLa cells were transfected with plasmids pEGFP or pEGFP::*rtxA1*_1491–1971_. F-actin was visualized using Alexa Fluor 594-conjugated phalloidin (Thermofisher Scientific, MA, USA). To visualize filamin A, HeLa cells were stained with a polyclonal anti-filamin A antibody (Santa Cruz Biotechnology, CA, USA) and an Alexa Fluor 594-conjugated anti-rabbit IgG secondary antibody (Thermofisher Scientific, MA, USA).

### Co-immunoprecipitation assay

HeLa, M2 (filamin-negative) and A7 (filamin-overexpression) cells grown in 100-mm tissue culture plates (Nalge Nunc International, New York, USA) were transiently transfected with pEGFP or pEGFP::*rtxA1*_1491–1971_ using Lipofectamine 3000 for 30 h. Next, cells were lysed with lysis buffer [50 mM Tris (pH 7.4), 150 mM NaCl, 1 mM EDTA, 1% Triton X-100, 10% glycerol, 10 μg/mL leupeptin, 10 μg/mL aprotinin, and 2 mM PMSF] on ice for 4 h. Cell lysates were centrifuged at 13,000 rpm for 10 min. The supernatants were subsequently collected and pre-incubated with immunopure immobilized protein A (Pierce) with gentle shaking at 4°C for 1 h to remove non-specific proteins bound to the beads. Subsequently, the supernatants (1 mg protein) were incubated with 1 µg of mouse monoclonal anti-filamin A (Santa Cruz Biotechnology, CA, USA) or 1 µg of rabbit polyclonal anti-GFP (Sigma-Aldrich, MO, USA) antibodies for 1 h, after which they were incubated with 20 µL of protein A agarose beads with gentle agitation at 4°C overnight. After centrifugation, pellets were washed with lysis buffer. The target proteins were detected by Western blotting using antibodies against filamin A (1:1000), actin (1:1000, Sigma-Aldrich, MO, USA), RtxA1_1491–1971_ (1:500), and GFP (1:4000).

In addition, HeLa cells were infected with *V. vulnificus* strains at an MOI of 100 for 1 h. The immunoprecipitates and lysates were blotted with antibodies against filamin A (1:1000), RtxA1_1491–1971_ (1:500) and actin (1:1000).

### Transfection with small interfering RNAs

The siRNAs against filamin A, pak1 and the negative control siRNA were purchased from Santa Cruz Biotechnology. HeLa cells were incubated for 1 day with DMEM supplemented with 10% FBS without antibiotics. Each siRNA (10 nM) was transfected into cells using Lipofectamine RNAiMAX (Thermofisher Scientific, MA, USA) according to the manufacturer’s instructions and were incubated for 2 days. Filamin A and pak1 knockdown was confirmed by Western blotting.

### LDH assay

The cytotoxicity of *V. vulnificus* toward host cells was measured using a CytoTox96 non-radioactive cytotoxicity assay kit (Promega, Madison, WI). HeLa, M2 (filamin-negative) and A7 (filamin-overexpression) cells seeded into 48-well plates were infected with *V. vulnificus* strains at an MOI of 100 for the indicated times. Lactate dehydrogenase (LDH) released in the supernatants was measured as a cytotoxicity parameter.

### Western blotting

Western blotting was carried out as the method described previously [22] with the appropriate primary antibodies against filamin A (1:1000), actin (1:1000), RtxA1_1491–1971_ (1:500), GFP (1:4000), GAPDH (1:1000, Thermofisher Scientific, MA, USA), and phospho-pak1, phospho-JNK or phospho-p38 (1:500, Cell Signaling Technology, MA, USA) at 4°C overnight, with a subsequently incubation with horseradish-peroxidase-conjugated second antibodies. Immunoreactive proteins were visualized using an ECL Western blot detection system (Advansta, Menlo Park, CA, USA).

### Statistical analysis

Results are expressed as the means ± SEM. Significant differences were evaluated using the Student *t*-test, with *P* values < 0.05 considered significant. All experiments were repeated at least three times, and the data were obtained at least in triplicate. Results shown are from the representative experiments.

## Results

### RtxA1_1491–1971_ of V. vulnificus causes HeLa cell rounding

**Figure 1.**
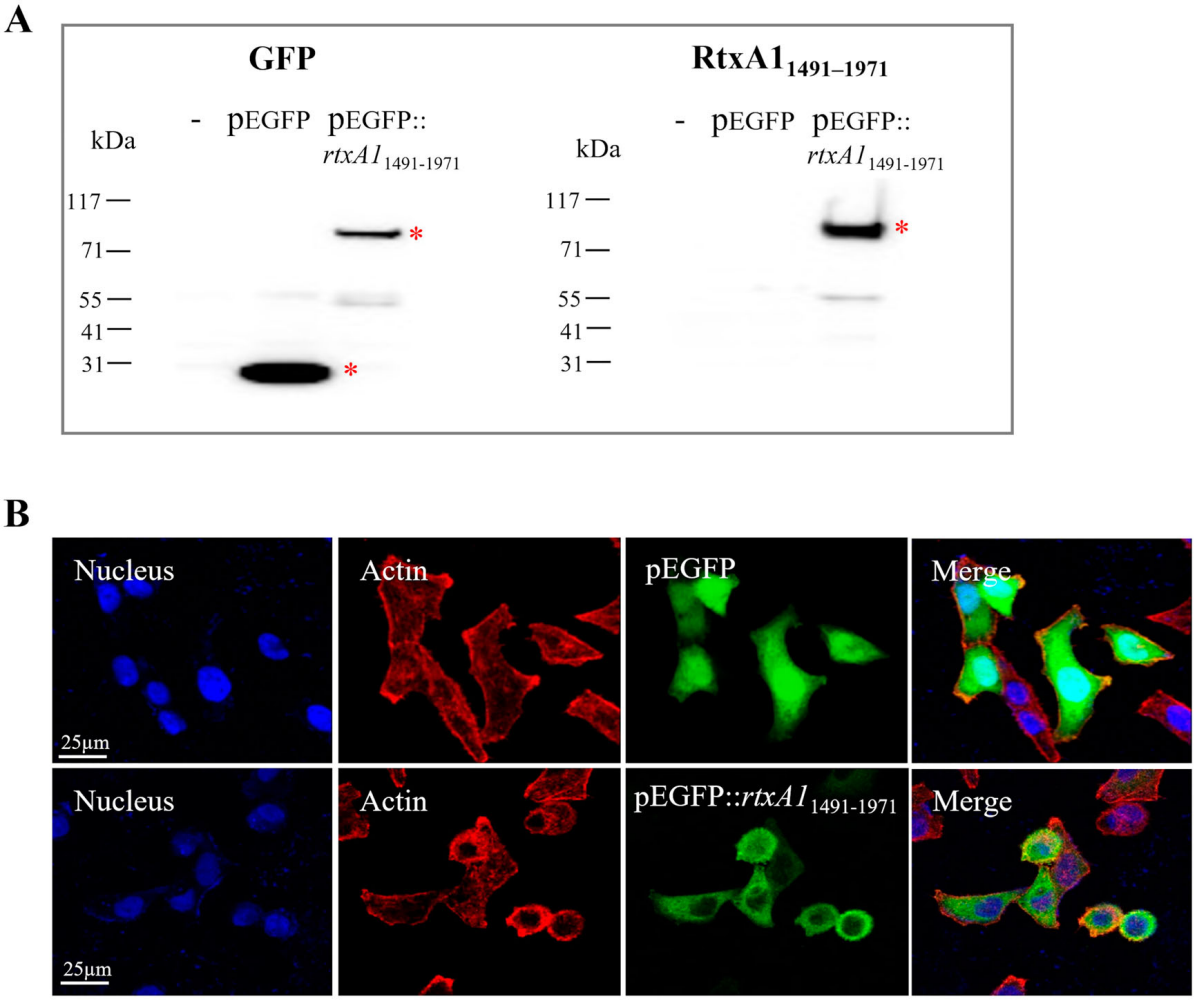
RtxA1_1491–1971_ of *V. vulnificus* causes HeLa cell rounding. HeLa cells were transfected with plasmids pEGFP or pEGFP::*rtxA1*_1491–1971_. (A) GFP or RtxA1_1491–1971_ proteins were detected by Western blotting. (B) Actin was visualized by staining with Alexa Fluor 594 conjugated phalloidin. Cells were mounted with an anti-fade reagent with DAPI. Fluorescence images were acquired using a laser scanning confocal microscope.

RtxA1_1491–1971_ was expressed as a GFP fusion protein in HeLa cells and was validated using antibodies specific to GFP and RtxA1_1491–1971_ (Figure 1(A)). HeLa cells transfected with pEGFP::*rtxA1*_1491–1971_ or with an empty pEGFP vector (green) were stained with Alexa Fluor 594 conjugated to phalloidin (red) and DAPI (blue) to visualize actin and nuclei, respectively. Confocal microscopic images revealed that EGFP::RtxA1_1491–1971_ expression was confined to the cytosol and RtxA1_1491–1971_-expressing cells tended to be rounded. Control EGFP expression was observed in all cells, with strong expression detected in the nuclei (Figure 1(B)).

### Filamin A is a host partner of RtxA1_1491–1971_

Host partners binding to RtxA1_1491–1971_ were screened using a yeast two-hybrid screening assay, resulting in the identification of several host proteins. Among them, filamin A (amino acids 1801–2017) was detected multiple times and was further studied as a candidate host partner of RtxA1_1491–1971_. Filamin binding to RtxA1_1491–1971_ was detected in the yeast screen by intense blue staining (Figure S1).

**Figure 2.**
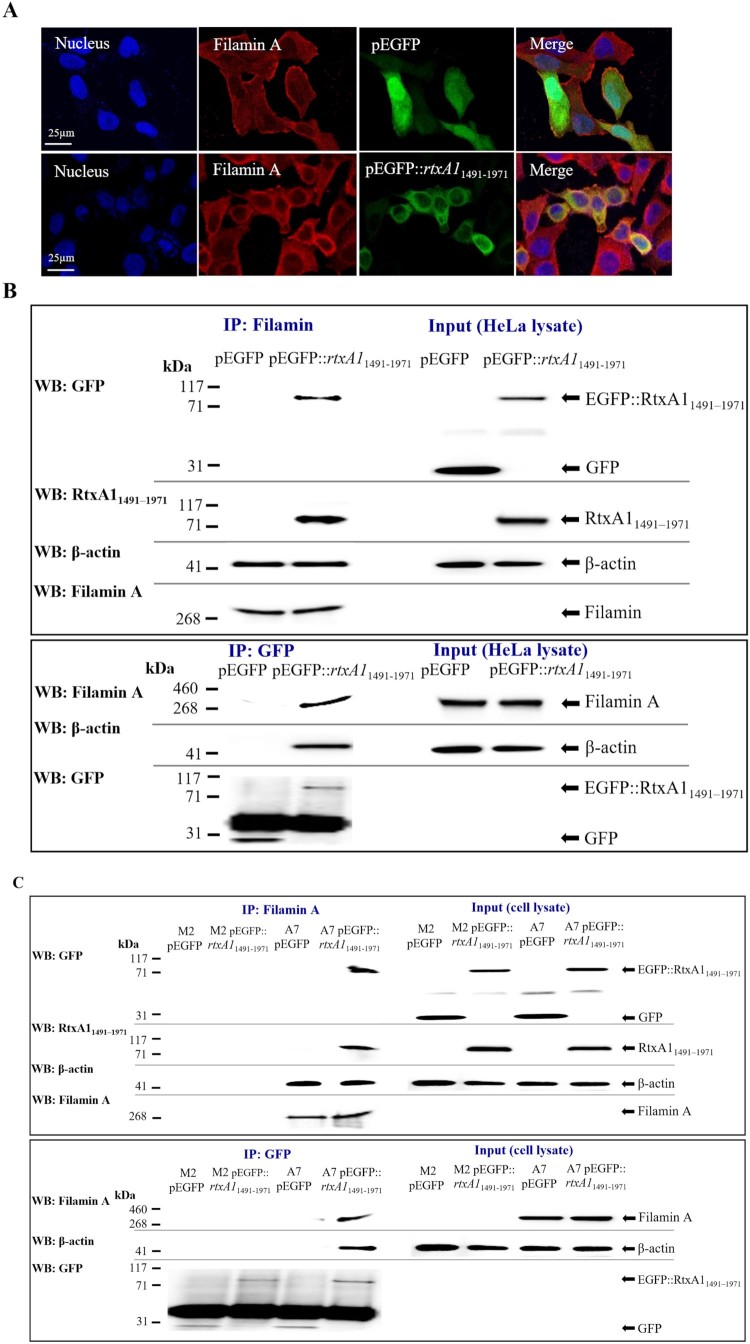
Filamin A is a host partner of RtxA1_1491–1971_. (A) HeLa cells were transfected with plasmids pEGFP or pEGFP::*rtxA1*_1491–1971_. Filamin A was stained with the anti-filamin A antibody and Alexa Fluor 594 conjugated anti-rabbit second antibody (red). Then cells were mounted with an anti-fade reagent with DAPI. Fluorescence images were acquired using a laser scanning confocal microscope. (B) HeLa cells and (C) M2 and A7 cells were transfected with pEGFP or pEGFP::*rtxA1*_1491–1971_. The immunoprecipitates and lysates were blotted with antibodies against GFP, RtxA1_1491–1971_, filamin A or actin (IP: immunoprecipitation, WB: Western blotting).

**Figure 3.**
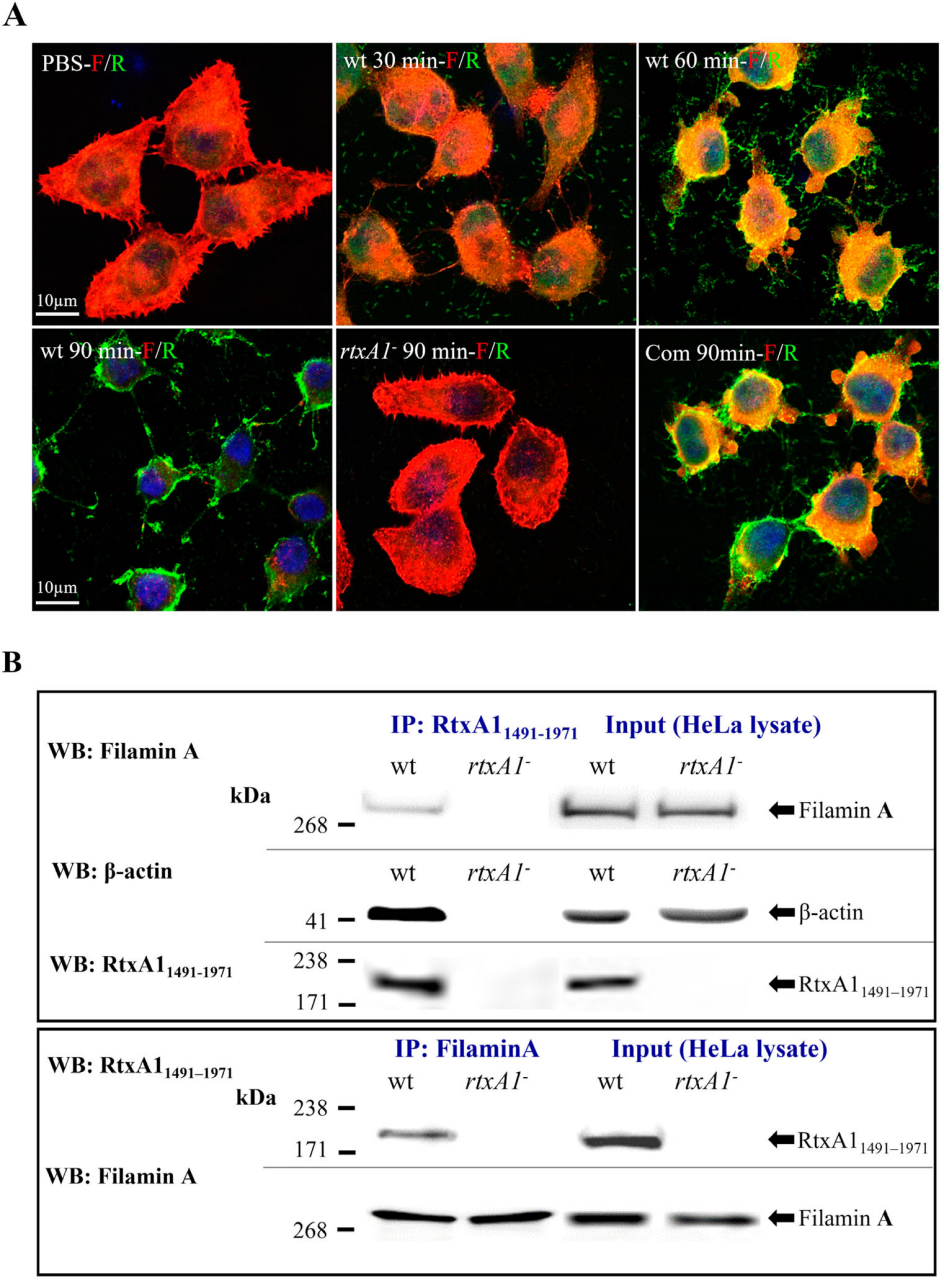
RtxA1 of *V. vulnificus* directly interacts with filamin A and promotes its aggregation. (A) HeLa cells were infected with *V. vulnificus* wt, *rtxA1^−^*, and the complement strain of *rtxA1^−^* (Com) at an MOI of 100 for indicated time at 37°C. RtxA1_1491–1971_ was stained with the primary antibody and then with a FITC-conjugated anti-rabbit IgG (green). Filamin A was stained with the primary antibody and then with a Texas Red-conjugated anti-mouse IgG (red). F: Filamin A (red); R: RtxA1_1491–1971_ (green). (B) HeLa cells were infected with bacteria at an MOI of 100 for 1 h. The immunoprecipitates and lysates were blotted with antibodies against filamin A, RtxA1_1491–1971_ or actin (IP: immunoprecipitation, WB: Western blotting).

HeLa cells transfected with pEGFP::*rtxA1*_1491–1971_ or with an empty pEGFP vector (green) were stained with an anti-filamin A antibody (red) and Alexa Fluor 594 conjugated secondary antibody. The colocalization of RtxA1_1491–1971_ with filamin A was observed via confocal microscopy, and RtxA1_1491–1971_-expressing cells tended to be rounded (Figure 2(A)). Co-IP using HeLa, M2 (filamin-negative) and A7 (filamin-overexpression) cells transfected with pEGFP or pEGFP::*rtxA1*_1491–1971_ was also performed, the results of which demonstrated co-immunoprecipitation of the RtxA1_1491–1971_ and filamin A (Figure 2(B), (C)). Interestingly, β-actin was also coprecipitated with filamin A and RtxA1_1491–1971_, suggesting binding between actin and filamin A (Figures 2(B), (C), 3(B)).

### RtxA1 of V. vulnificus directly interacts with filamin A and promotes its aggregation

HeLa cells treated with *V. vulnificus* wt promoted the dramatic aggregation of filamin A, and the tight, globular filamin A aggregates were removed from the cytoplasm by a unique pinching-off mechanism. Filamin A appeared to be depleted over time in HeLa cells infected with *V. vulnificus* wt, but remained intact in cells infected with an *rtxA1* mutant. Furthermore, the mutant phenotype was fully complemented in the complement strain in trans with the *V. vulnificus* wt allele encoded by a plasmid (Figure 3(A)). The levels of actin and filamin A in cell pellets and supernatants were also detected by Western blot analysis. Both proteins could be detected in the cell pellets and appeared in the supernatants from 75 min after infection. After 90 min of infection, the two cytoskeletal proteins could not be detected in the cell pellets. Interestingly, the level of GAPDH, a cytosolic control protein used as an internal control for the analysis, did not change significantly in the infected cell pellets*.* This result suggested that the cytoskeletal proteins were specifically released from cells intoxicated with RtxA1 (Figure S2(A)). To determine whether only cytoskeletal proteins were translocated or if other intracellular proteins or organelles were also released, we monitored the levels of α-tubulin, EGFR, TomB, GAPDH, and nucleophosmin, representing proteins associated with the cytoskeleton, plasma membrane, mitochondria, cytosol, and the nucleus of HeLa cells infected with *V. vulnificus* strains. With the exception of the cytoskeletal proteins actin, α-tubulin and filamin A, none of the proteins tested were detected in the culture supernatants of HeLa cells infected with *V. vulnificus* wt. The translocation of cytoskeletal proteins was not observed in HeLa cells infected with *V. cholerae* N16961, suggesting that the depletion of cytoskeletal proteins is a unique characteristic of the *V. vulnificus* RtxA1 protein compared to the *V. cholerae* MARTX toxin (Figure S2(B)). The domain was also immunoprecipitated with protein lysates from HeLa cells infected with *V. vulnificus* at an MOI of 100 for 1 h. Polyclonal RtxA1_1491–1971_ and monoclonal filamin A antibodies reciprocally coprecipitated RtxA1_1491–1971_ and filamin A (Figure 3(B)).

### V. vulnificus RtxA1-induced cytotoxicity and activation of the JNK and p38 MAPKs are regulated by filamin A

**Figure 4.**
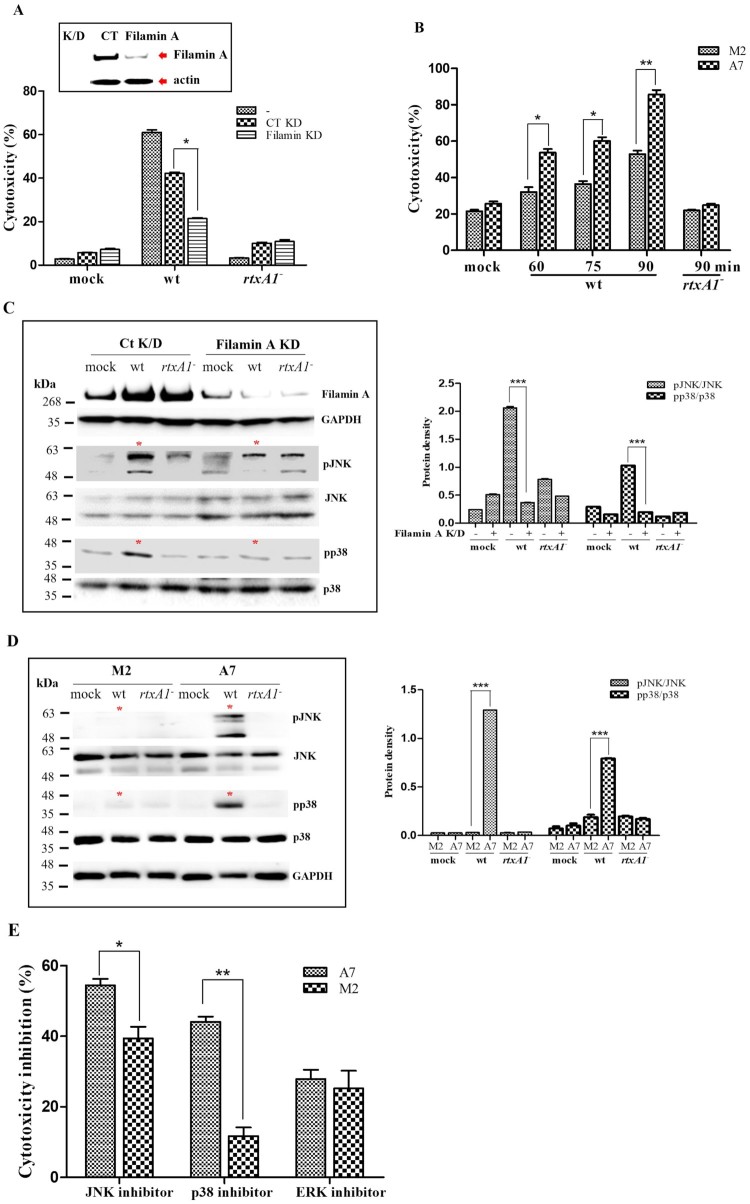
*V. vulnificus* RtxA1-induced cytotoxicity and activation of the JNK and p38 MAPKs are regulated by filamin A. (A) Filamin A KD was conducted in HeLa cells with siRNA and was confirmed by Western blotting, with actin used as the loading control. Filamin A-KD HeLa cells were infected with bacteria at an MOI of 100 for 90 min. LDH released in the supernatants was assayed. (B) M2 and A7 cells were infected with bacteria at an MOI of 100. LDH released in the supernatants was assayed. (C) Filamin A-KD HeLa cells and (D) M2 and A7 cells were infected with bacteria at an MOI of 100 for 50 min. Cell lysates were used for Western blotting. Protein levels from three independent experiments were quantified using Image J software. (E) M2 and A7 cells pretreated with MAPK inhibitors for 1 h were infected with bacteria at an MOI of 100 for 90 min, after which LDH released in the supernatants was assayed. Results are expressed as means ± SEM, and statistical significance was defined as *P < 0.05, **P < 0.01, ***P < 0.001.

To study the role of filamin A in RtxA1-mediated cytotoxicity, filamin A was knocked down by transfecting HeLa cells with small interfering RNA (siRNA) against filamin A, which was confirmed by Western blotting. A marked reduction in filamin A expression was observed after the transfection of cells with filamin A-specific siRNA, and the downregulation of filamin A expression in host cells resulted in a significant decrease in RtxA1-mediated cytotoxicity (Figure 4(A)). We also evaluated the RtxA1-mediated cytotoxicity in M2 (filamin-negative) and A7 (filamin-overexpression) cells, the latter of which exhibited more significant cytotoxicity than the filamin-negative M2 cells (Figure 4(B)).

MAPKs are well-known to serve as signalling proteins that elicit cellular responses to infecting pathogens. In our previous study, the JNK and p38 MAPKs appeared to play significant roles in the RtxA1-mediated necrotic death of HeLa cells [3]. We hypothesized that MAPK signalling may be controlled by the RtxA1_1491–1971_-filamin A interaction. To investigate the role of filamin A in *V. vulnificus*-mediated MAPK activation, the phosphorylation of JNK and p38 promoted by RtxA1 was examined in filamin A-knockdown HeLa cells. The results showed that the phosphorylation of both MAPKs was significantly decreased in filamin A-knockdown HeLa cells (Figure 4(C)). Filamin A-mediated MAPK phosphorylation induced by RtxA1 was also confirmed in M2 (filamin-negative) and A7 (filamin-overexpression) cells. However, the phosphorylation of JNK and p38 was not observed during the RtxA1 intoxication of filamin A-deficient M2 cells compared with filamin-transfected subline A7 cells, suggesting that the RtxA1-filamin A interaction mediates MAPK signalling in RtxA1-intoxicated host cells (Figure 4(D)).

In addition, we also tested whether the RtxA1-mediated activation of MAPKs involves filamin using pharmacological antagonists. SP600125 (10 µM, a JNK antagonist) and SB203580 (10 µM, a p38 MAPK inhibitor) showed greater inhibitory effects in A7 (filamin-overexpression) cells than M2 (filamin-negative) cells on the *V. vulnificus* RtxA1-mediated cytotoxicity (Figure 4(E)).

### Pak1 regulates RtxA1-dependent activation of the JNK and p38 MAPKs through filamin A

**Figure 5.**
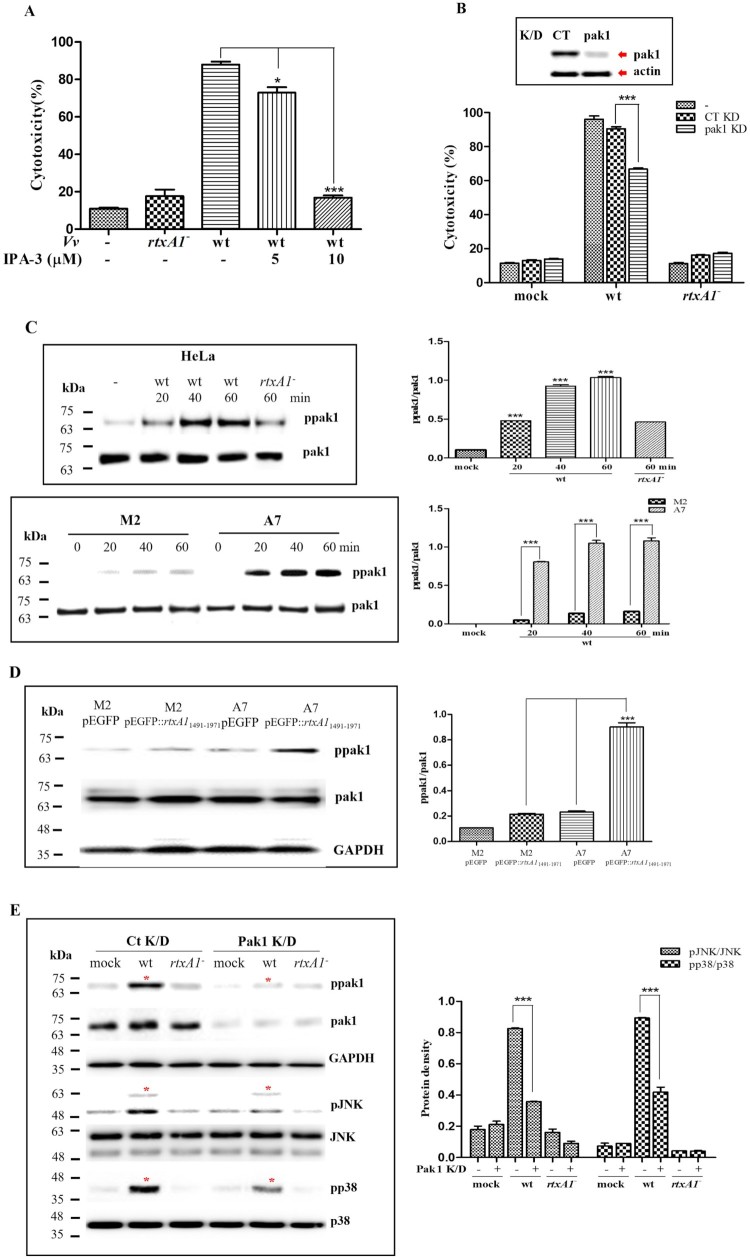
Pak1 regulates RtxA1-dependent activation of the JNK and p38 MAPKs through filamin A. (A) HeLa cells pretreated with IPA-3 for 1 h and (B) Pak1-KD HeLa cells were infected with bacteria at an MOI of 100 for 90 min, and cytotoxicity was analyzed by LDH assay. (C) HeLa, M2 and A7 cells were infected with bacteria at an MOI of 100. Cell lysates were used for Western blotting. (D) M2 and A7 cells were transfected with plasmids pEGFP or pEGFP::*rtxA1*_1491–1971_, and cell lysates were used for Western blotting. (E) Pak1-KD HeLa cells were infected with bacteria at an MOI of 100 for 50 min. Cell lysates were used for Western blotting. Pak1 KD was confirmed by Western blotting, and GAPDH was used as the loading control. Protein levels from three independent experiments were quantified using Image J software. Results are expressed as means ± SEM, and statistical significance was defined as *P < 0.05, ***P < 0.001.

Filamin A is known to be a cytoprotective protein, and its mechanoprotective activity is associated with its ability to act as scaffolding factor by recruiting multiple kinases, such as MKK4 and pak1 [23,24]. We observed that IPA-3, a pak1 inhibitor suppressed RtxA1-induced cytotoxicity (Figure 5(A)), and the downregulation of pak1 also decreased the cytotoxicity caused by *V. vulnificus* wt (Figure 5(B)). As shown in Figure 5(C), HeLa, M2 and A7 cells were infected with *V. vulnificus* wt, and we found that RtxA1 toxin induced the phosphorylation of pak1 in a time dependent manner in the presence of filamin. To further identify that the co-expression of RtxA1_1491–1971_ and filamin A induced the phosphorylation of pak1, M2 and A7 cells were transfected with plasmids of pEGFP or pEGFP::*rtxA1*_1491–1971_. The result showed that only A7 cells (filamin-overexpression) transfected with pEGFP::*rtxA1*_1491–1971_ showed the remarkably higher expression levels on the phosphorylation of pak1 (Figure 5(D)). These results showed that RtxA1-activated pak1 is involved in the interactions of RtxA1_1491–1971_ fragment with the host factor filamin A. The Pak family of serine/threonine kinases are involved in multiple cellular process, including cytoskeletal reorganization, MAPK signalling, and apoptosis signalling [25–27]. To investigate the role of pak1 in *V. vulnificus*-mediated MAPK activation, the phosphorylation of JNK and p38 promoted by RtxA1 was examined in pak1-knockdown HeLa cells. Results showed that the phosphorylation of both MAPKs significantly decreased in pak1-knockdown HeLa cells (Figure 5(E)). These results suggest that the RtxA1_1491–1971_-filamin A interaction triggers pak1 phosphorylation to induce the MAPK signalling responsible for cytoskeletal rearrangement-mediated host cell rounding and cytotoxicity.

### RtxA1-induced cytoskeletal arrangement is mediated by the activities of the pak1, JNK, and p38 kinases

**Figure 6.**
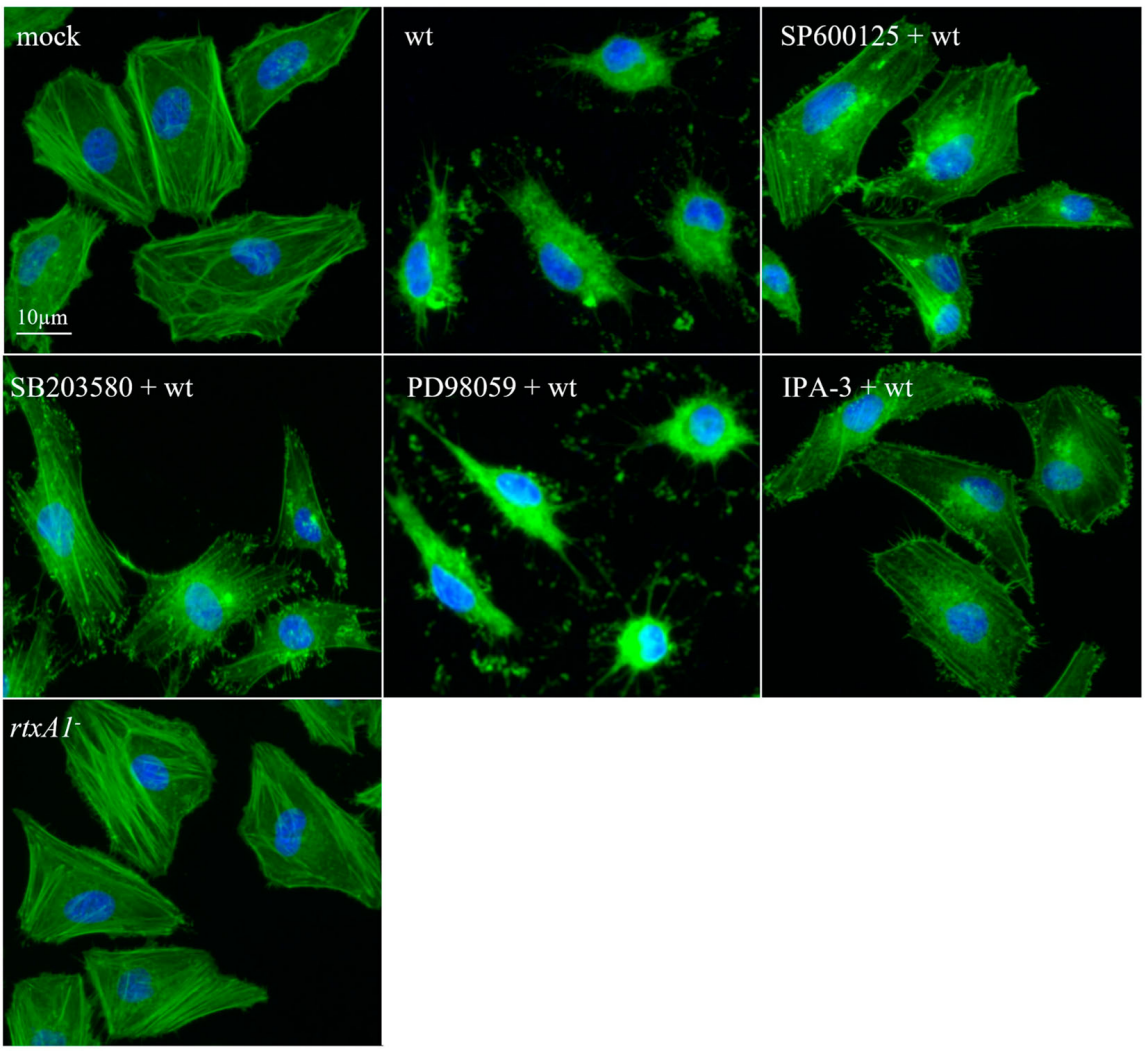
Effects of kinase inhibitors on RtxA1-induced cytoskeletal rearrangement. HeLa cells pretreated with inhibitors of MAPKs or pak1 were infected with bacteria at an MOI of 100 for 40 min. Actin was visualized by staining with Alexa Fluor 488 conjugated phalloidin. Cells were then mounted with an anti-fade reagent with DAPI. Fluorescence images were acquired using a fluorescence microscope.

We also visualized the effects of kinase inhibitors on RtxA1-induced cytoskeletal arrangement in *V. vulnificus*-infected HeLa cells. As shown in Figure 6, *V. vulnificus* RtxA1 toxin caused cell rounding and actin aggregation, which were inhibited by the pretreatment of cells with pharmacological antagonists, such as SP600125 (10 µM, a JNK antagonist), SB203580 (10 µM, a p38 MAPK inhibitor), and IPA-3 (10 µM, a pak1 inhibitor), but not PD98059 (10 µM, an ERK inhibitor).

## Discussion

*V. vulnificus* RtxA1 toxin plays dominant roles in *in vivo* pathogenesis and acute host cell death, and in the early stage of *V. vulnificus*-induced cytotoxicity, RtxA1 causes host cell rounding [3,7]. Interestingly, we found HeLa cells transfected with the plasmid pEGFP::*rtxA1*_1491–1971_ exhibited cell rounding. The putative *V. vulnificus* RtxA1_1491–1971_ domain, corresponding to the ERM domain as shown in previous study [28], shows 88% identity with the corresponding domain located in the N-terminal conserved domain of *V. cholerae* MARTX. This domain showed ∼25% homology to ERM family proteins, which link the plasma membrane to cortical actin cytoskeleton in a well-regulated manner [29]. A previous study reported that the actin cytoskeleton is hijacked to promote HIV entry into its target cells, and ERM, filamin and cofilin have been shown to play crucial roles in this process [30]. To assess the structural homology of the RtxA1_1491–1971_ domain with other known ERM domain-containing proteins, we performed a structural alignment of the RtxA1_1491–1971_ domain with the FERM domain of the Merlin protein encoded by the *M. musculus Nf-2* gene (pdb file downloaded from https://www.ebi.ac.uk/pdbe/entry/pdb/1isn/, green), for which the 3D structure has been solved by X-ray crystallography [31]. The results revealed structural homology between the N-terminal regions of the proteins (aa 38–48 of RtxA1_1491–1971_ and 67–131 of the FERM domain of Merlin; yellowed lines) (Figure S3). This region is known to have a membrane-association function through direct binding with the cytoplasmic region of integral membrane proteins [32]. This result suggested that RtxA1_1491–1971_ may associate with the inner leaflet of the cytoplasmic membrane in a similar fashion to other ERM family proteins. The remaining regions of RtxA1_1491–1971_ may interact with filamin A and play roles in activating MAPKs, which should be investigated in future structural biology studies.

Furthermore, we demonstrated the occurrence of specific interactions between RtxA1_1491–1971_ and filamin A through microscopic observations and co-IP, which confirmed the direct binding of RtxA1_1491–1971_ and filamin A. Interestingly, *V. vulnificus* RtxA1 appeared to mediate the release of filamin A along with other cytoskeletal proteins, such as actin and α-tubulin, from infected HeLa cells, while retaining other organelle marker proteins in intoxicated cells (Figures 1–3 and S2). Filamin A, which was identified in 1975 as the first discovered non-muscle actin filament cross-linking protein, is capable of stabilizing three-dimensional actin filament networks [33] and serves as a scaffold protein for over 90 binding partners, including channels, receptors, intracellular signalling molecules, and even transcription factors [34]. Given the versatile functions of filamin, we speculated filamin A may play significant roles in both cytoskeletal rearrangement and cytotoxicity caused by *V. vulnificus* RtxA1. The downregulation of filamin A expression decreased the cytotoxicity of *V. vulnificus* (Figure 4(A), (B)), suggesting that the RtxA1-filamin interaction provides a scaffold for signalling proteins involved in the RtxA1-mediated programmed necrotic cell death. In addition, the JNK and p38 MAPK signalling pathways were observed to be downstream of the RtxA1-filamin A interaction during the cell death process (Figure 4(C), (D)). In our previous studies, we showed that MAPK signalling is involved in the RtxA1-mediated programmed necrosis of host cells [3]. Since filamin A is located beneath the cell membrane and serves as an anchor for actin filaments, the RtxA1_1491–1971_ domain should be flipped inside to interact with filamin A to trigger cytoskeletal rearrangement and MAPK signalling. In the signalling pathway, pak1 appears to have an important role on filamin A-associated downstream MAPK activation and cytotoxicity (Figure 5). Several mechanisms that induce pak activity have been reported, and the binding of GTPase Rac/cdc42 to Pak causes autophosphorylation and conformational changes in Pak [35]. However, many of the effects of pak1 on the actin cytoskeleton appear to be independent of GTPase activity but dependent on protein–protein interactions [36]. Pak1 is localized in regions of the cortical actin cytoskeleton [37], and filamin A-binding to pak1 stimulates both the autophosphorylation and its kinase activity [36]. These findings suggest that pak1 plays a central role in inducing actin rearrangements and MAPK-mediated host cell responses during the RtxA1-mediated programmed necrotic cell death process. Other RtxA1 domains also appear to be involved in the MAPK activation pathway [38]. From the results of this study, it is evident that pak1 phosphorylation, resulting from RtxA1_1491–1971_-filamin interaction, is the upstream of MAPK signalling and that inhibition of this signalling axis significantly inhibits host cell death.

To the best of our knowledge, this is the first report on the role of filamin A in the cell-killing mechanism of a bacterial cytotoxin. The results of a limited number of studies have indicated the role of filamins in bacterial pathogenesis. During *Ureaplasma parvum* infection, the phosphorylation of filamin A occurs in response to various cell signalling cascades that regulate cell motility, differentiation, apoptosis, and inflammation [39]. The VopV protein from *Vibrio parahaemolyticus* induces marked rearrangement of the apical epithelial cell membrane, including the elimination of microvilli, through interactions with actin and filamin [40]. Filamin A has been also reported to regulate the actin-dependent clustering of HIV receptors [41]. Given the important scaffolding roles of filamin A in the normal physiology of eukaryotic cells, there may be additional filamin A-targeting microbial/parasitic virulence factors that modulate cytoskeletal arrangements and MAPK signalling pathways.

## Supplementary Material

Supplemental Material
