# Supplementary material for: Vibrio vulnificus RtxA1 cytotoxin targets filamin A to regulate PAK1- and MAPK-dependent cytoskeleton reorganization and cell death
Source: Emerg Microbes Infect. 2019 Jun 25;8(1):934–45. doi: 10.1080/22221751.2019.1632153 (PMC6598492; doi:10.1080/22221751.2019.1632153)
Supplement: Supplemental Material [file TEMI_A_1632153_SM6816.docx]

**Supplementary materials**

**Figure S1**


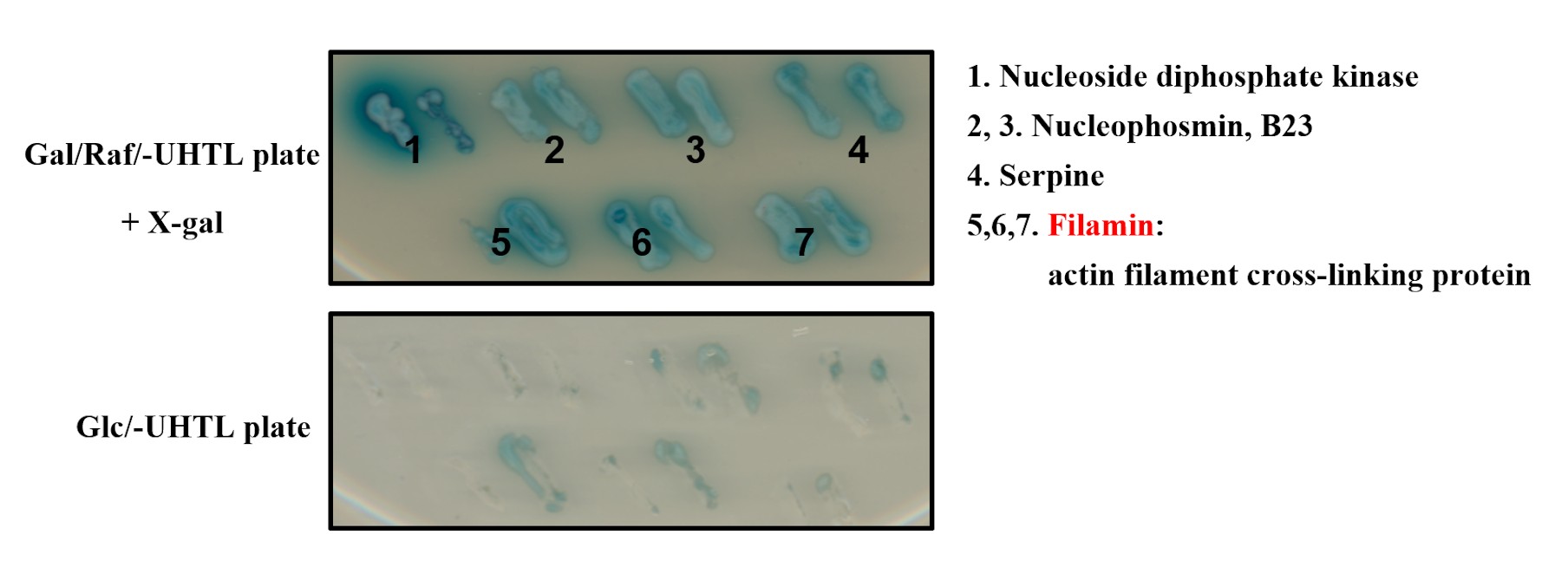


**Figure S2**


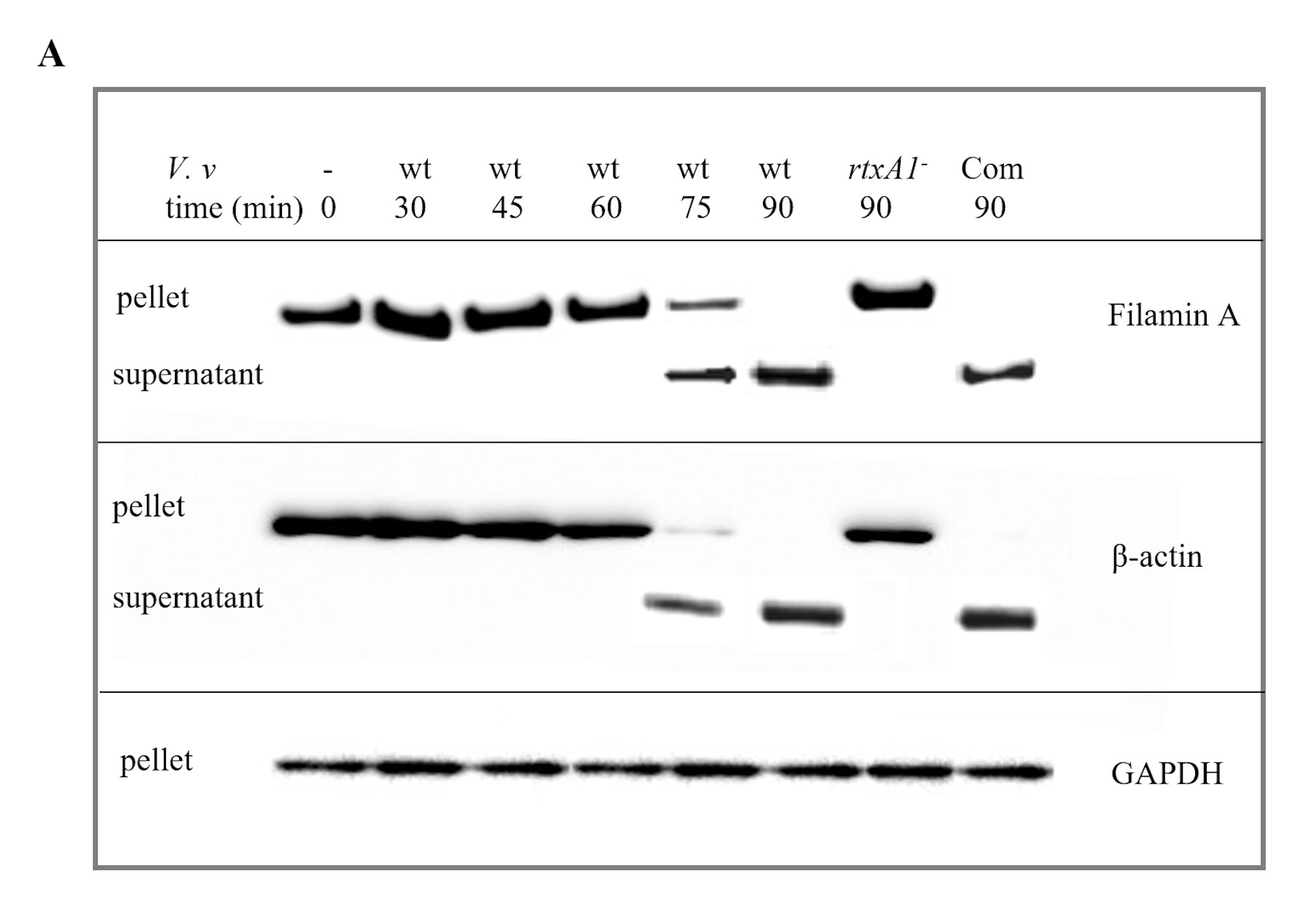

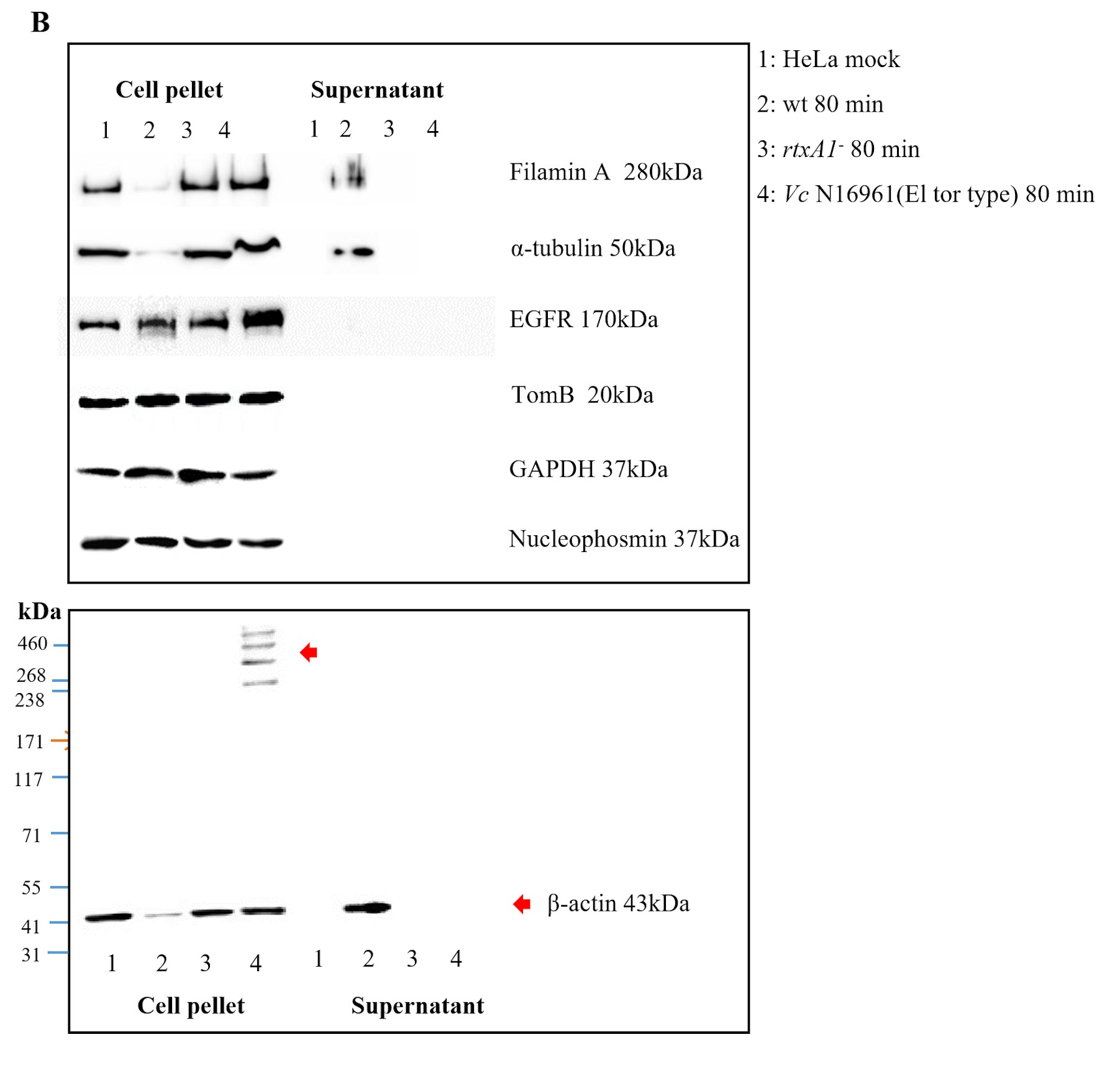


**Figure S3**


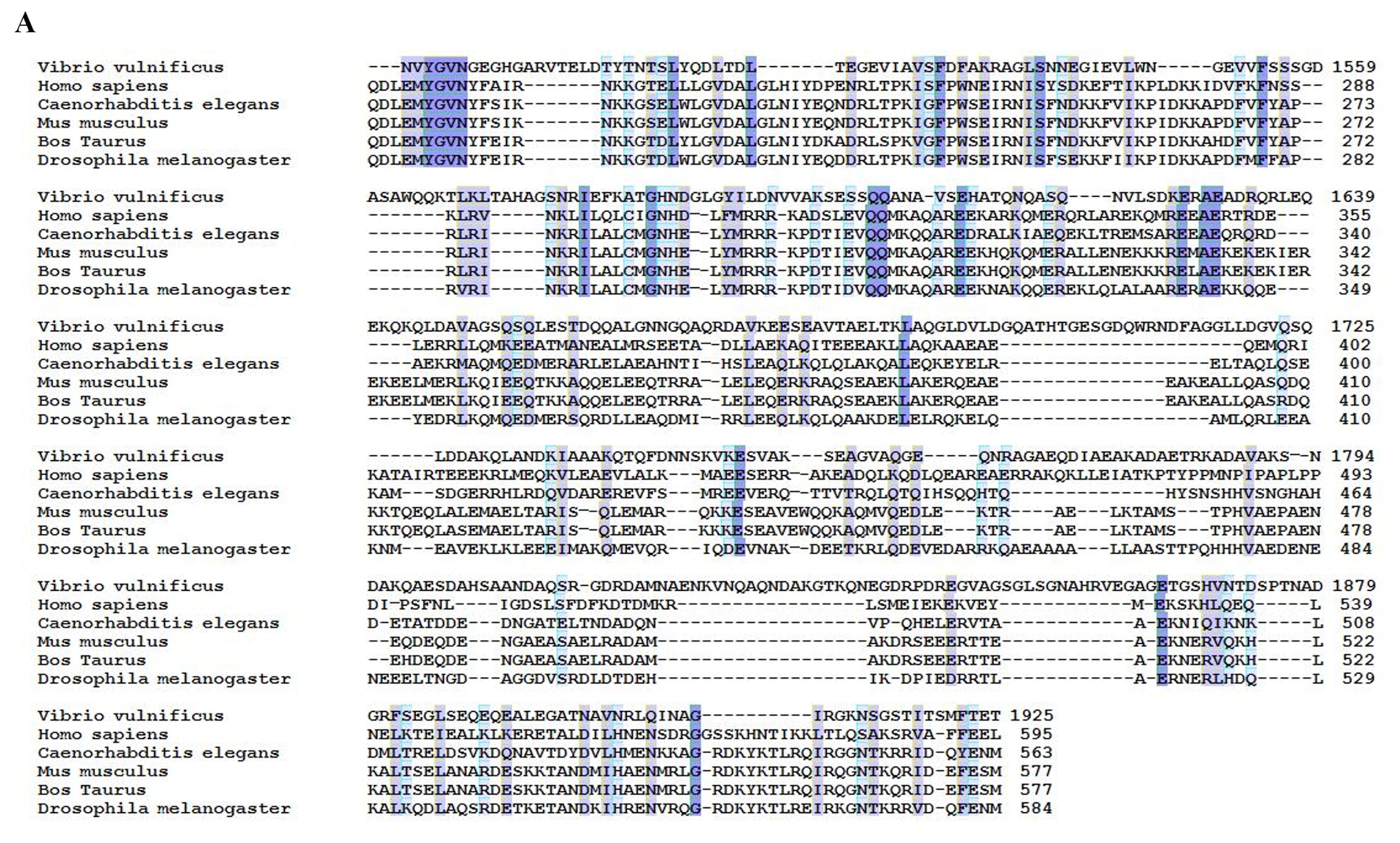

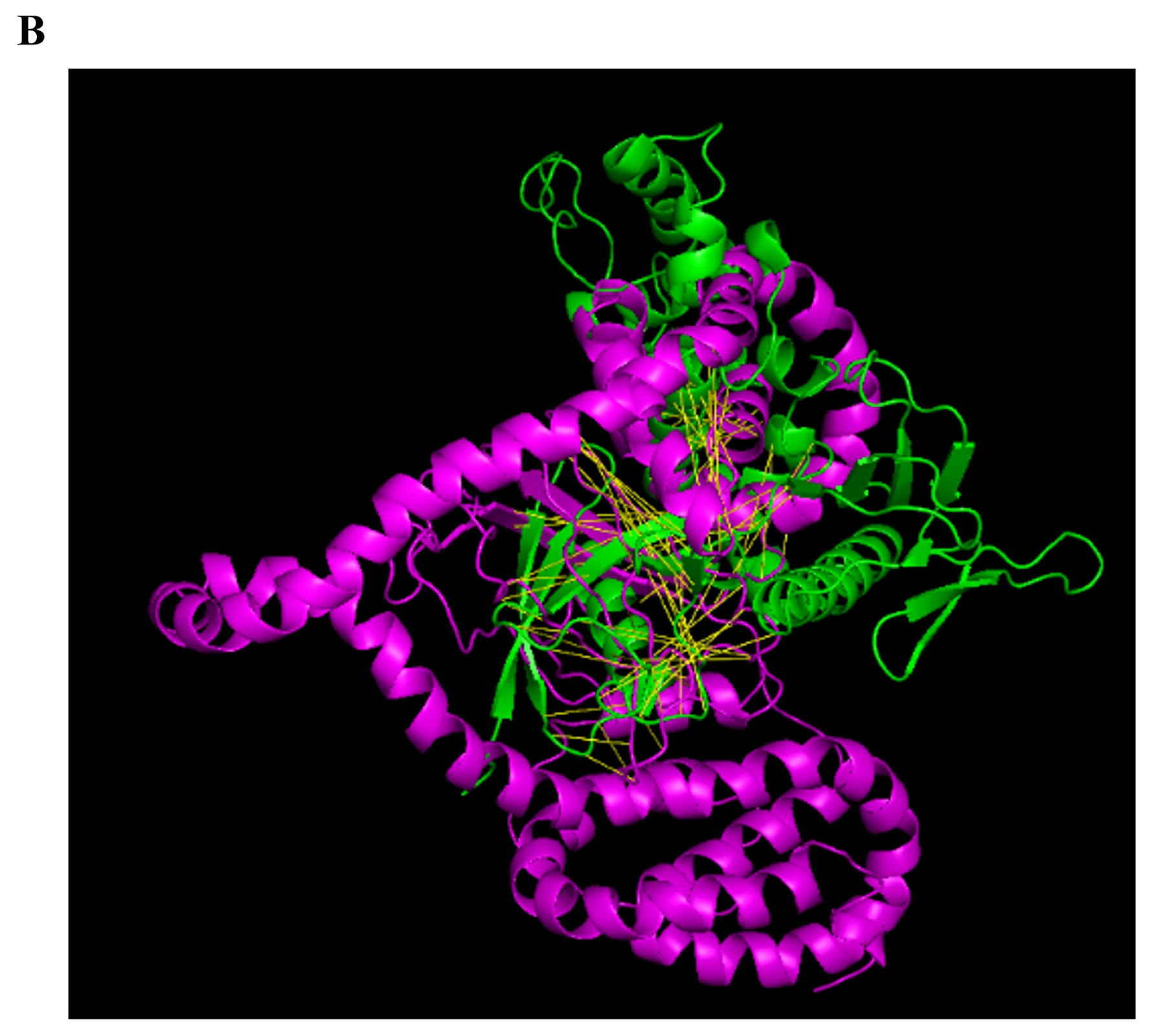


**Figure Legends**

**Figure S1.** Yeast two-hybrid screen for host interaction partners of RtxA1_1491–1971_. The DNA fragment encoding RtxA1_1491–1971_ was cloned into the vector pLexA and the resulting bait plasmid was used to screen a HeLa cDNA library fused to the vector pYESTrp2. Yeast cells (EGY48/p8op-lacZ) were used for the two-hybrid assay. Double transformants were selected on inducing galactose/raffinose complete medium minus Uracil (Ura), tryptophan (Trp), leucine (Leu), and histidine (His) plates containing X-galactosidase [(Gal/Raf/-UHTL X-gal) and noninducing (Glc/-UHTL) to test reporter gene expression. Filamin binding to RtxA1_1491–1971_ was detected in the yeast screen demonstrated by intense blue staining. This experiment was repeated multiple times with similar results.

**Figure S2.** (A) HeLa cells were infected with wt or *rtxA1^-^* strains at an MOI of 100 for indicated times. HeLa cell lysates and the precipitants of 300 μL culture supernatants were used for Western blotting of filamin A and actin. GAPDH was used as the loading control (Com: the complement strain of *rtxA1^-^*). (B) HeLa cells were infected with wt, *rtxA1^-^* or *Vc* N16961 at an MOI of 100 for 80 min. Host cell organelle marker proteins in the HeLa cell lysates and the culture supernatants were detected using specific antibodies to filamin A, actin, GAPDH, -tubulin, EGFR, TomB, or nucleophosmin by Western blot analysis. Data are representative of at least three independent experiments.

**Figure S3.** RtxA1_1491–1971_ domain shows structural homology with FERM domain. (A) Polypeptide sequence alignment of *V. vulnificus* RtxA1_1491–1971_ with ERM proteins. The amino acid sequences of *V. vulnificus* RtxA1_1491–1971_ were compared with ERM-like protein from *H. sapiens*, ERM from *C. elegans*, moesin from *M. musculus*, moesin from *B. Taurus*, and a moesin isoform from *D. melanogaster* (corresponding protein accession numbers: WP_058645630.1, AAA36212.1, CCD62462.1, AAH47366.1, NP_001039942.1, and NP_001259361.1, respectively). Conserved residues are shaded; the darker the color, the more conserved the residue is. (B) Structural alignment of the RtxA1_1491–1971_ with the 1isn (FERM) domain of the *Nf-2* gene of *M. musculus* (pdb downloaded from https://www.ebi.ac.uk/pdbe/entry/ pdb/1isn/, green), of which the 3D structure was reported by X-ray crystallography. The RtxA1_1491–1971_ 3D structure was simulated by using the Phyre2 protein fold recognition algorithm (<http://www.sbg.bio.ic.ac.uk/> phyre2/html/). For the structural alignment, MacPyMol Ver. 1.8 (<http://pymol.org)> was used. Data are representative of at least three independent experiments.
